# Divergence of allosteric effects of rapacuronium on binding and function of muscarinic receptors

**DOI:** 10.1186/1471-2210-9-15

**Published:** 2009-12-28

**Authors:** Jan Jakubík, Alena Randáková, Esam E El-Fakahany, Vladimír Doležal

**Affiliations:** 1 Institute of Physiology, Academy of Sciences of the Czech Republic, Prague, Czech Republic; 2 Division of Neuroscience Research in Psychiatry, University of Minnesota Medical School, Minneapolis, MN 55455, USA

## Abstract

**Background:**

Many neuromuscular blockers act as negative allosteric modulators of muscarinic acetylcholine receptors by decreasing affinity and potency of acetylcholine. The neuromuscular blocker rapacuronium has been shown to have facilitatory effects at muscarinic receptors leading to bronchospasm. We examined the influence of rapacuronium on acetylcholine (ACh) binding to and activation of individual subtypes of muscarinic receptors expressed in Chinese hamster ovary cells to determine its receptor selectivity.

**Results:**

At equilibrium rapacuronium bound to all subtypes of muscarinic receptors with micromolar affinity (2.7-17 μM) and displayed negative cooperativity with both high- and low-affinity ACh binding states. Rapacuronium accelerated [^3^H]ACh association with and dissociation from odd-numbered receptor subtypes. With respect to [^35^S]GTPγS binding rapacuronium alone behaved as an inverse agonist at all subtypes. Rapacuronium concentration-dependently decreased the potency of ACh-induced [^35^S]GTPγS binding at M_2 _and M_4 _receptors. In contrast, 0.1 μM rapacuronium significantly increased ACh potency at M_1_, M_3_, and M_5 _receptors. Kinetic measurements at M_3 _receptors showed acceleration of the rate of ACh-induced [^35^S]GTPγS binding by rapacuronium.

**Conclusions:**

Our data demonstrate a novel dichotomy in rapacuronium effects at odd-numbered muscarinic receptors. Rapacuronium accelerates the rate of ACh binding but decreases its affinity under equilibrium conditions. This results in potentiation of receptor activation at low concentrations of rapacuronium (1 μM) but not at high concentrations (10 μM). These observations highlight the relevance and necessity of performing physiological tests under non-equilibrium conditions in evaluating the functional effects of allosteric modulators at muscarinic receptors. They also provide molecular basis for potentiating M_3 _receptor-mediated bronchoconstriction.

## Background

Five subtypes of muscarinic acetylcholine receptors that belong to class A of G-protein coupled receptors have been identified [1]. The primary response of stimulation of the M_2 _and M_4 _subtypes of muscarinic receptors is activation of the G_i/o _class of G-proteins resulting in inhibition of adenylyl cyclase, whereas stimulation of M_1_, M_3_, and M_5 _receptors leads to activation of the G_q/11 _class of G-proteins and stimulation of phospholipase C[2]. Muscarinic receptors mediate many diverse physiological functions that are selectively mediated by different receptor subtypes [3]. This is why discovery of selective ligands is of prime importance for clinical practice. However, due to the very conserved nature of the orthosteric binding site of muscarinic acetylcholine receptors the selectivity of orthosteric agonists is very poor [4]. Orthosteric antagonists that bind to less conserved amino acids located close to the orthosteric binding site display better selectivity than orthosteric agonists. Muscarinic allosteric ligands exhibit remarkable selectivity among receptor subtypes [5]. They interact mainly with the second and the third extracellular loops that are much less conserved than transmembrane segments creating the orthosteric binding site [6-10].

The extraordinary selectivity of allosteric modulators that is due to differences in both affinity and cooperativity [11] has attracted attention of pharmacologists in the past decade. Somewhat paradoxically, most of originally discovered and probably best studied allosteric compounds of muscarinic receptors are neuromuscular blockers [12-14]. By definition, these are competitive nicotinic acetylcholine receptor antagonists but many of them have high affinities and strong allosteric interactions, particularly at the M_2 _subtype of muscarinic receptors.

In clinical practice, different competitive (nondepolarizing) neuromuscular blockers are employed to induce muscle relaxation to facilitate intubation during surgery. The neuromuscular blocker rapacuronium was withdrawn from clinical use due to high incidence of bronchospasm resulting in death [15]. Parasympathetic innervation of airways transmits signal via postsynaptic M_3 _receptors that mediate acetylcholine-induced contraction and M_2 _receptors that inhibit with high potency smooth muscle relaxation mediated by increase in cytoplasmic cAMP [16]. M_2 _receptors are also located at parasympathetic cholinergic nerve terminals innervating smooth muscle and their stimulation inhibits acetylcholine (ACh) release [17]. In functional experiments on the guinea pig trachea preparation it was demonstrated that rapacuronium preferentially antagonizes M_2 _over M_3 _muscarinic receptors [18]. In addition, involvement of allosteric potentiation of ACh binding to muscarinic M_3 _receptors in bronchospasm induced by rapacuronium was suggested, but not proven [19]. A very recent paper confirmed a unique behavior of rapacuronium compared to other skeletal muscle relaxants in vivo and demonstrated that rapacuronium potentiates bronchoconstriction evoked by both naturally released and exogenous acetylcholine, indicating an important role of postsynaptic M_3 _receptors [20].

Because we have been interested in investigations of positive cooperativity of allosteric ligands with ACh binding [11,21] and allosteric agonists [22] these findings led us to analyze in detail the interactions of rapacuronium with acetylcholine binding and receptor activation of all subtypes of muscarinic receptors heterologously expressed in membranes of Chinese hamster ovary (CHO) cells. We demonstrate that rapacuronium binds to and exhibits negative cooperativity with ACh binding at all subtypes of muscarinic receptors. Surprisingly, low concentrations of rapacuronium potentiate ACh-induced signaling at the M_1_, M_3_, and M_5 _receptor subtypes and accelerate ACh binding. This striking behavior is unparallel at other neuromuscular blockers.

## Results

**Figure 1:**
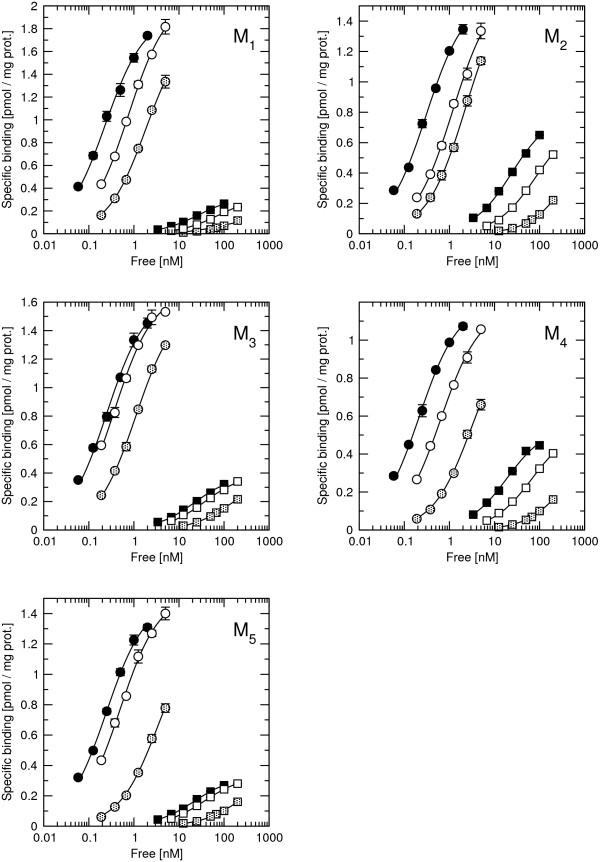
**Saturation binding of [^3^H]NMS and [^3^H]Ach**. Specific binding of [^3^H]NMS (circles) and [^3^H]ACh (squares) to membranes from CHO cells expressing individual subtypes of muscarinic receptors is plotted against the concentration of free radioligand. Binding of radioligand in the absence (closed symbols) and presence of 10 μM (open symbols) or 100 μM (hatched symbols) rapacuronium, respectively. Data are means ± SE from 3 independent experiments performed in quadruplicates. Curves are fits of Eq. 1 to data. Binding parameters are summarized in Table 1.

**Figure 2:**
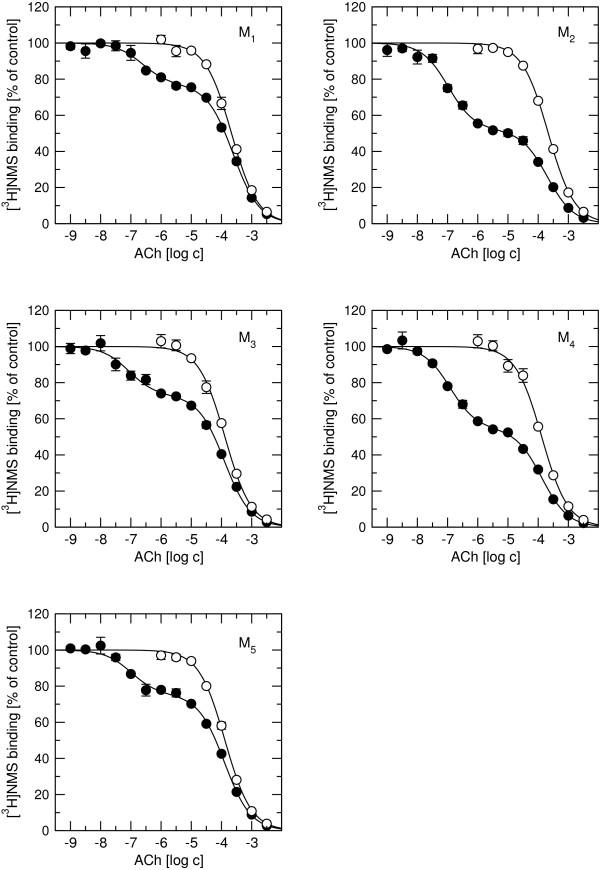
**Effects of 10 μM GTPγS on ACh competition with [^3^H]NMS binding**. Binding of 1 nM [^3^H]NMS to the membranes from CHO cells expressing individual subtypes of muscarinic receptors in the absence (closed circles) or presence (open circles) of 1 μM GTPγS is expressed as per cent of control binding and are plotted against concentration of ACh. Data are means ± SE of 3 independent experiments performed in quadruplicates. Binding parameters are described in the Results.

Saturation binding experiments (Figure 1; Table 1) with 68 pM to 2 nM [^3^H]NMS in cell membranes showed similar binding capacity (1 to 2 pmol of binding sites per mg of protein) and affinity (equilibrium dissociation constant (K_D_) ranging from 205 pM at M_4 _to 320 pM at M_2 _receptors) for all receptor subtypes (Figure. 1; Table 1). Significant depletion (up to 34% at M_1 _for 68 pM [^3^H]NMS) occurred despite the use of 0.8 ml incubation volume in the binding assays. Thus, free concentrations of [^3^H]NMS were calculated and used in Eq. 1. Saturation binding experiments with 3.4 nM to 100 nM [^3^H]ACh showed similar high affinity binding among all subtypes with K_D _around 20 nM. Rapacuronium concentration dependently decreased affinity for [^3^H]NMS and [^3^H]ACh at all subtypes without change in maximum binding capacity (B_MAX_). Competition experiments of unlabeled ACh vs. [^3^H]NMS displayed high and low binding sites for ACh at all subtypes with higher proportion of high affinity binding sites at even-numbered subtypes (Figure 2). Equilibrium dissociation constants (K_I_) of ACh high-affinity binding derived from competition experiments with [^3^H]NMS (pK_D _= 7.32 ± 0.06, 7.59 ± 0.03, 7.79 ± 0.05, 7.69 ± 0.04, 7.68 ± 0.05, mean ± SE for M_1 _to M_5 _receptor) correspond to those measured in [^3^H]ACh saturation experiments (Table 1). In the presence of 10 μM GTPγS to uncouple receptors and G-proteins ACh low affinity binding was similar at all five subtypes with equilibrium dissociation constant (K_I_) ranging from 25.5 μM at M_4 _to 46.8 μM at M_1_.

**Table 1:** Effects of rapacuronium on [^3^H]NMS and [^3^H]ACh saturation binding

	pK_D_	B_MAX_	pK_D_'	B_MAX_	pK_D_'	B_MAX_
	**[^3^H]NMS**		**+ 10 μM rapacuronium**		**+ 100 μM rapacuronium**	

M_1_	9.60 ± 0.04	1.98 ± 0.20	9.14 ± 0.03*	2.03 ± 0.15	8.73 ± 0.03*	1.85 ± 0.17
M_2_	9.49 ± 0.03	1.56 ± 0.16	8.92 ± 0.04*	1.62 ± 0.14	8.64 ± 0.03*	1.66 ± 0.14
M_3_	9.64 ± 0.05	1.64 ± 0.17	9.44 ± 0.03*	1.67 ± 0.14	8.96 ± 0.03*	1.59 ± 0.16
M_4_	9.69 ± 0.04	1.19 ± 0.14	9.19 ± 0.02*	1.17 ± 0.13	8.52 ± 0.06*	1.06 ± 0.10
M_5_	9.59 ± 0.03	1.49 ± 0.16	9.31 ± 0.02*	1.53 ± 0.15	8.46 ± 0.06*	1.33 ± 0.13

	**[^3^H]Ach**		**+ 10 μM rapacuronium**		**+ 100 μM rapacuronium**	

M_1_	7.58 ± 0.05	0.33 ± 0.05	7.12 ± 0.03*	0.33 ± 0.05	6.43 ± 0.05*	0.32 ± 0.05
M_2_	7.63 ± 0.03	0.80 ± 0.08	7.09 ± 0.05*	0.74 ± 0.07	6.30 ± 0.06*	0.77 ± 0.08
M_3_	7.67 ± 0.05	0.39 ± 0.05	7.43 ± 0.04*	0.40 ± 0.04	6.85 ± 0.03*	0.37 ± 0.05
M_4_	7.69 ± 0.04	0.55 ± 0.04	7.17 ± 0.03*	0.54 ± 0.05	6.41 ± 0.05*	0.47 ± 0.04
M_5_	7.68 ± 0.03	0.33 ± 0.06	7.43 ± 0.02*	0.33 ± 0.03	6.64 ± 0.04*	0.34 ± 0.06

Negative logarithms of equilibrium dissociation constants (pK_D_) and maximum binding capacities (B_MAX _in fmol/μg of protein) of radioligands were obtained from saturation experiments shown in Figure 1 by fitting Eq. 1 to the data. Values are means ± SE of fits to 3 independent experiments performed in quadruplicates.

*P < 0.05; significantly different from control (radioligand alone) by ANOVA and Tukey-Kramer post-test.

**Figure 3:**
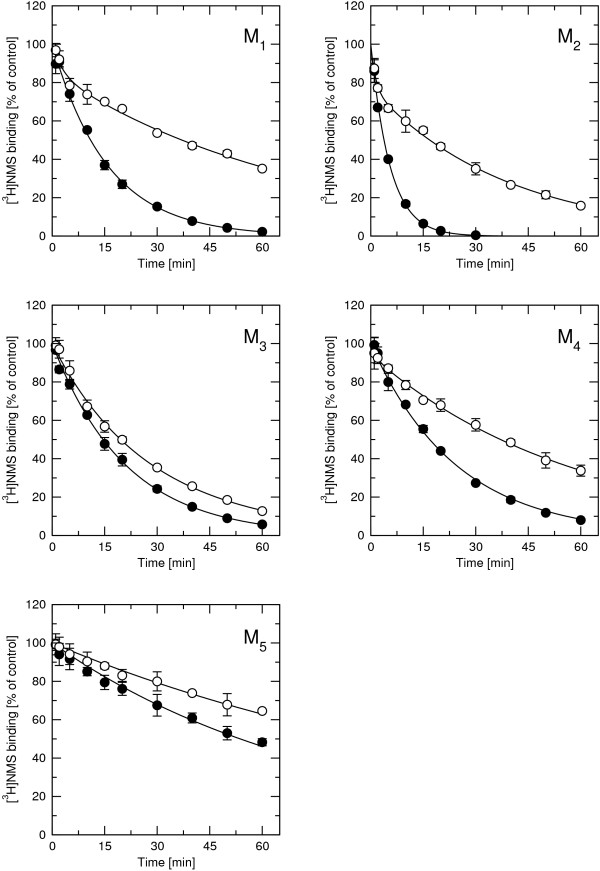
**Effects of 100 μM rapacuronium on dissociation of [^3^H]NMS binding**. Binding of [^3^H]NMS to membranes from CHO cells expressing individual subtypes of muscarinic receptors at different times after the addition of 10 μM NMS (closed circles) or a mixture of 10 μM NMS and 100 μM rapacuronium (open circles). Specific binding is expressed as percent of binding at time 0. Data are means ± SE from 3 independent experiments performed in quadruplicates. Binding parameters are summarized in Table 2.

Effects of 100 μM rapacuronium on the rate of ([^3^H]NMS) dissociation were measured in membranes from CHO cell expressing individual subtypes of muscarinic receptors after 60 min preincubation with 1 nM [^3^H]NMS. Dissociation was evoked by addition of 10 μM unlabeled NMS. Rapacuronium slowed dissociation of [^3^H]NMS from all subtypes of muscarinic ACh receptors (Figure 3, Table 2). This is an established hallmark of allosteric receptor modulation. It had the strongest effect at M_2 _receptors (7-fold decrease in rate of dissociation) and weakest effect at M_3 _and M_5 _receptors (40% decrease). While dissociation evoked by NMS was monophasic (Figure 3 closed symbols) it became biphasic in the presence of 100 μM rapacuronium with the exception of the M_5 _subtype.

**Table 2:** Effects of rapacuronium on the rate of [^3^H]NMS dissociation.

	Control	+ 100 μM rapacuronium		
	k_off _[min^-1^]	k_off1 _[min^-1^]	f_2 _[%]	k_off2 _[min^-1^]
M_1_	0.063 ± 0.004	0.014 ± 0.001*	14 ± 3	0.34 ± 0.05
M_2_	0.18 ± 0.01	0.026 ± 0.002*	23 ± 5	0.76 ± 0.11
M_3_	0.048 ± 0.003	0.031 ± 0.002*	20 ± 4	0.058 ± 0.009
M_4_	0.041 ± 0.002	0.017 ± 0.001*	6.0 ± 2.0	0.75 ± 0.11
M_5_	0.013 ± 0.001	0.0078 ± 0.0004*		

Observed rates of [^3^H]NMS dissociation (k_off_, k_off1 _and k_off2_) and fraction of sites (f_2_) with faster dissociation (k_off2_) from individual subtypes of muscarinic receptors were obtained by fitting Eq. 7a and 7b to data in Figure 3. Results of better fit are shown. Values are means ± SE of fits to 3 independent experiments performed in quadruplicates. *P < 0.05; significantly different from control ([^3^H]NMS alone) by t-test

**Figure 4:**
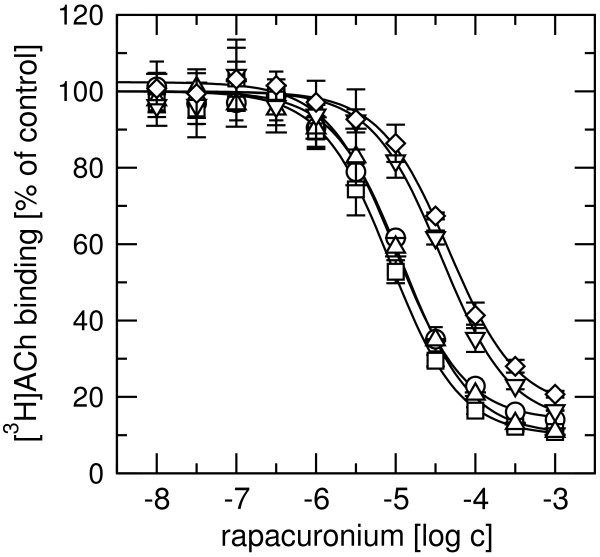
**Effects of rapacuronium on high-affinity [^3^H]ACh binding**. Binding of 20 nM [^3^H]ACh to membranes from CHO cells expressing individual subtypes of muscarinic receptors (circles, M_1_; squares, M_2_; diamonds, M_3_; up-triangles, M_4_; down-triangles, M_5_) was determined in the presence of rapacuronium at the concentrations indicated on the x-axis and is expressed as percent of specific binding in the absence of rapacuronium. Data are means ± SE from 3 independent experiments performed in quadruplicates. Curves are fits of Eq. 3 to data. Binding parameters are summarized in Table 3.

**Figure 5:**
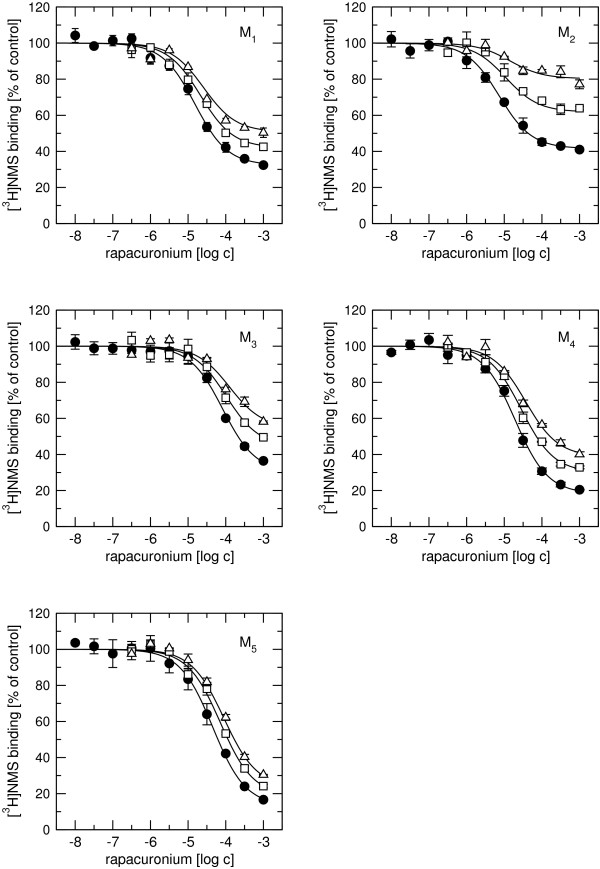
**Effects of rapacuronium on low-affinity ACh binding and [^3^H]NMS binding**. Binding of 1 nM [^3^H]NMS to membranes from CHO cells expressing individual subtypes of muscarinic receptors was measured in the presence of rapacuronium at the concentrations indicated on the x-axis. Data are expressed as percent of specific binding in the absence of rapacuronium. Symbols represent binding of [^3^H]NMS (circles), and [^3^H]NMS in the presence of 10 μM GTPγS and 100 μM (squares) or 200 μM (triangles) ACh. Binding of [^3^H]NMS was decreased by 100 μM ACh to 71, 64, 59, 60 and 56% and by 200 μM ACh to 55, 47, 42, 43 and 39% at M_1 _to M_5 _receptors, respectively. Bigger divergence of curves denotes stronger negative cooperativity between ACh and rapacuronium binding. Data are means ± SE from 3 independent experiments performed in quadruplicates. Curves are fits of Eq. 3 (circles) and Eq. 4 (squares and triangles) to data prior to normalization. Binding parameters are summarized in Table 3.

Displacement radioligand binding experiments with either 20 nM [^3^H]ACh (Figure 4) or 1 nM [^3^H]NMS (Figure 5, circles) and increasing concentrations of rapacuronium showed that rapacuronium binds equally well to all five muscarinic receptor subtypes. Equilibrium dissociation constants (pK_A_; Table 3) for rapacuronium derived from experiments with [^3^H]NMS and [^3^H]ACh were virtually the same with a rank order of affinity of M_2_>M_4_>M_1_>M_5_>M_3 _(range from 2.6-17.8 μM). Rapacuronium displayed negative cooperativity with [^3^H]NMS in binding to all subtypes, as evidenced by a maximal limit to its effects on the affinity of the radioligand that differed as a function of radioligand concentration. These effects were strongest at the M_5 _subtype (35-fold decrease in affinity) and weakest at the M_2 _subtype (6.8-fold decrease in affinity; Figure 5, closed circles). While cooperativity of rapacuronium with high- (pα) and low-affinity (pβ) ACh binding was essentially the same at individual subtypes (Table 3, row-wise comparison) it was slightly different among subtypes (e.g. 16-fold decrease in ACh low-affinity binding at M_3 _receptors vs. 36-times decrease at M_4 _receptors; Table 3, column-wise comparison).

**Table 3:** Binding parameters of NMS, ACh and rapacuronium to membranes from CHO cells expressing M_1 _through M_5 _receptor subtypes.

	[^3^H]NMS		[^3^H]Ach		Ach	
			high affinity binding		low affinity binding	
	pK_A_	pα	pK_A_	pα	pK_A_	pβ
M_1_	5.37 ± 0.03	-1.08 ± 0.05	5.37 ± 0.04	-1.30 ± 0.07	5.33 ± 0.03	-1.32 ± 0.06
M_2_	5.59 ± 0.03	-0.83 ± 0.07	5.55 ± 0.05	-1.46 ± 0.08	5.56 ± 0.04	-1.52 ± 0.06
M_3_	4.75 ± 0.04	-1.11 ± 0.05	4.80 ± 0.04	-1.26 ± 0.05	4.77 ± 0.05	-1.20 ± 0.07
M_4_	5.42 ± 0.04	-1.40 ± 0.04	5.41 ± 0.05	-1.51 ± 0.05	5.49 ± 0.04	-1.56 ± 0.07
M_5_	4.97 ± 0.04	-1.54 ± 0.05	4.92 ± 0.03	-1.34 ± 0.07	4.95 ± 0.04	-1.28 ± 0.07

Negative logarithm of equilibrium dissociation constant of rapacuronium (pK_A_) and factor of cooperativity (α ) between rapacuronium and radioligand ([^3^H]NMS or [^3^H]ACh, respectively) binding were obtained by fitting Eq. 3 to the data in Figures 4 and 5. Negative logarithm of equilibrium dissociation constant of rapacuronium (pK_A_) and factors of cooperativity (β) between rapacuronium and acetylcholine low affinity binding were obtained by fitting Eq. 4 to the data in Figure 5. Factors of cooperativity α and β are expressed as negative logarithms so that negative values represent negative cooperativity. Values are means ± SE of fits to 3 independent experiments performed in quadruplicates.

**Figure 6:**
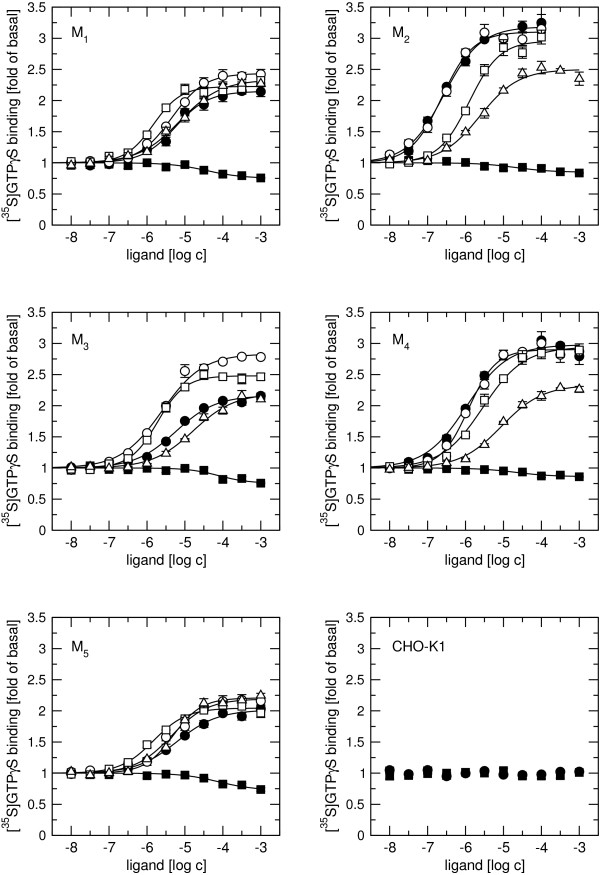
**Effects of rapacuronium on ACh-induced [^35^S]GTPγS binding to membranes**. Membranes from CHO cells expressing individual subtypes of muscarinic receptors or nontransfected CHO-K1 cells were preincubated for 60 min with buffer (closed symbols) or with rapacuronium (open symbols). [^35^S]GTPγS binding was induced by rapacuronium alone (closed squares), ACh alone (closed circles), or by ACh in the presence of rapacuronium (0.1 μM (open circles), 1 μM (open squares), or 10 μM (open triangles)). Data are expressed as fold increase of basal [^35^S]GTPγS binding and are presented as means ± SE from 3 independent experiments performed in quadruplicates. Curves are fits of Eq. 5 to data. Parameters are summarized in Tables 4 and 5.

Rapacuronium alone concentration dependently lowered [^35^S]GTPγS binding to membranes (Figure 6, closed squares; Table 4) with a maximal effect of approximately 25% at odd-numbered subtypes and 15% at even-numbered subtypes, with similar half-effective concentrations (EC_50_) ranging from 28 μM at M_2 _receptors to 76 μM at M_3 _receptors. While the EC_50 _values of rapacuronium in inhibiting [^35^S]GTPγS binding at individual subtypes correlated with affinities measured in binding experiments with [^3^H]ACh (R^2 ^= 0.76) they were lower (4- to 12-fold) at all subtypes. We could not test the involvement of muscarinic receptors in the effects of rapacuronium on [^35^S]GTPγS binding using orthosteric antagonists, since 10 μM NMS or 10 μM atropine by themselves decreased [^35^S]GTPγS binding by more than 30% at all receptor subtypes. Inhibitory effects of rapacuronium were not additive to those of NMS or atropine (not shown). However, rapacuronium did not decrease [^35^S]GTPγS binding in membranes from nontransfected CHO cells (Figure 6, bottom row right).

**Table 4:** Direct effects of rapacuronium on [^35^S]GTPγS binding

	M_1_	M_2_	M_3_	M_4_	M_5_
pEC_50_	4.30 ± 0.04	4.55 ± 0.04	4.12 ± 0.03	4.44 ± 0.04	4.21 ± 0.04
E_MAX_	0.76 ± 0.08	0.84 ± 0.06	0.75 ± 0.07	0.86 ± 0.06	0.75 ± 0.07

Negative logarithms of half effective concentrations (pEC_50_) and E_MAX _of rapacuronium on [^35^S]GTPγS binding were obtained by fitting Eq. 5 to the data from the measurements of [^35^S]GTPγS binding in the presence of rapacuronium in concentrations ranging from 10^-8 ^to 10^-3 ^M normalized to the absence of rapacuronium (Figure 6, open squares). Values are means ± SE of fits to 3 independent experiments performed in quadruplicates.

As expected, ACh concentration-dependently stimulated [^35^S]GTPγS binding to membranes from cells expressing all individual subtypes of muscarinic receptors (Table 4 and Figure 6, closed circles). The maximal effect of ACh (E_MAX_) was about two-fold increase in basal binding at odd-numbered receptors and three-fold increase at even-numbered receptors with a rank order of efficacy of M_2_>M_4_>M_1_>M_5_>M_3 _(range from 3.12 to 1.99-fold increase). In control conditions ACh EC_50 _values were lower at even-numbered subtypes than at odd-numbered subtypes with a rank order of potency of M_2_>M_4_>M_3_>M_5_>M_1 _(range from 0.25 to 6.31 μM) (Table 5). While the EC_50 _of ACh-stimulated [^35^S]GTPγS binding was less than that of its low-affinity binding conformation by 178-times at M_2 _and 23-times at M_4 _receptors it was only 7.4-, 5.4-, and 4.7-times lower at M_1_, M_3_, and M_5 _receptors, respectively. In comparison with its high-affinity binding, the EC_50 _of ACh-stimulated [^35^S]GTPγS binding was only 10-times higher at M_2 _and 55-times at M_4 _receptors but 130-260- and 300-times higher at M_1_, M_5 _and M_3 _receptors, respectively. E_MAX _was about two-fold increase in basal binding at odd-numbered receptors and three-fold increase at even-numbered receptors with a rank order of efficacy of M_2_>M_4_>M_1_>M_5_>M_3 _(range from 3.12 to 1.99-fold increase) (Table 5).

**Table 5:** Effects of rapacuronium on ACh-stimulated [^35^S]GTPγS binding

	Acetylcholine		+ 0.1 μM rapacuronium		+ 1 μM rapacuronium		+ 10 μM rapacuronium	
	pEC_50_	E_MAX_	pEC_50_	E_MAX_	pEC_50_	E_MAX_	pEC_50_	E_MAX_
M_1_	5.20 ± 0.05	2.23 ± 0.08	5.41 ± 0.04*	2.35 ± 0.12	5.78 ± 0.04*	2.28 ± 0.12	5.22 ± 0.05	2.27 ± 0.11
M_2_	6.61 ± 0.03	3.12 ± 0.12	6.65 ± 0.03	3.10 ± 0.15	5.92 ± 0.05*	2.93 ± 0.13	5.67 ± 0.07*	2.46 ± 0.18*
M_3_	5.31 ± 0.05	2.12 ± 0.08	5.65 ± 0.04*	2.78 ± 0.11*	5.66 ± 0.04*	2.52 ± 0.08*	4.83 ± 0.05*	2.12 ± 0.09
M_4_	5.95 ± 0.04	2.93 ± 0.15	5.93 ± 0.04	2.95 ± 0.11	5.59 ± 0.05*	2.80 ± 0.12	5.12 ± 0.05*	2.32 ± 0.16*
M_5_	5.26 ± 0.05	1.99 ± 0.08	5.45 ± 0.04*	2.10 ± 0.09	5.82 ± 0.04*	2.04 ± 0.09	5.29 ± 0.05	2.22 ± 0.09

Negative logarithms of half effective concentrations (pEC_50_) and maximum stimulatory effect (E_MAX_) of acetylcholine on [^35^S]GTPγS binding in the absence or presence of the indicated concentrations of rapacuronium were obtained by fitting Eq. 5 to the data in Figure 6. Values are means ± SE of fits to 3 independent experiments performed in quadruplicates. *P < 0.05; significantly different from control (Ach alone) by ANOVA and Tukey-Kramer post-test.

Measurements of ACh-stimulated [^35^S]GTPγS binding in the presence of 0.1, 1 and 10 μM rapacuronium showed differential effects of rapacuronium on receptor activation by an orthosteric agonist at individual receptor subtypes (Figure 6 open symbols). At even-numbered subtypes 1 μM and 10 μM rapacuronium significantly increased ACh EC_50_, with lowering of E_MAX _at 10 μM rapacuronium. These results are in line with the effects of rapacuronium on ACh binding. In contrast, the effects of rapacuronium on activation of odd-numbered subtypes were more complex. At these subtypes rapacuronium had the strongest effect on activation of the M_3 _subtype. At this subtype 0.1 and 1 μM rapacuronium caused a significant 2-fold decrease in ACh EC_50 _and approximately 60% and 35% increase in E_MAX_, respectively. Rapacuronium at 10 μM increased ACh EC_50 _by about 3-fold without a significant change in E_MAX_. Rapacuronium (0.1 - 10 μM) had no effect on ACh efficacy at the M_1 _and M_5 _subtypes but decreased the EC_50 _of ACh in stimulating [^35^S]GTPγS binding by 1.5- and 4-fold, respectively, at concentrations of 0.1 and 1 μM. However, this effect was not evident at 10 μM rapacuronium (Figure 6).

**Figure 7:**
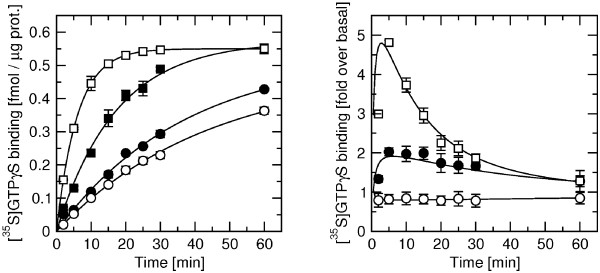
**Effects of rapacuronium on kinetics of [^35^S]GTPγS binding**. Membranes were preincubated for 60 min in the presence (open symbols) or absence (closed symbols) of 1 μM rapacuronium. Then [^35^S]GTPγS was added simultaneously with buffer (circles) or 10 μM ACh (squares). Incubations were terminated at the times indicated on the x-axis. The increase of specific [^35^S]GTPγS binding is expressed as fmol per μg of protein (left) and as fold increase of specific binding under basal conditions (right). Data are means ± SE of values from 3 independent experiments performed in quadruplicates.

Kinetics of [^35^S]GTPγS binding to membranes from CHO-M_3 _cells (where rapacuronium has the most pronounced effects) were measured after 5 min preincubation with 5 μM GDP and 60 min with 1 μM rapacuronium followed by simultaneous addition of [^35^S]GTPγS and ACh, with or without rapacuronium (Figure 7). Under basal conditions ([^35^S]GTPγS + buffer, Figure 7, closed circles) [^35^S]GTPγS bound to membranes with an observed rate constant (k_obs_) of 0.0269 ± 0.0022 min^-1^. Ten μM ACh accelerated the rate of [^35^S]GTPγS binding to 0.0583 ± 0.047 min^-1 ^(Figure 7, closed squares) and 1 μM rapacuronium further increased the rate to 0.166 ± 0.013 min^-1 ^(Figure 7, open squares). While 10 μM ACh alone produced only 2-fold increase in [^35^S]GTPγS binding at 5 min incubation it caused nearly 5-fold increase in the presence of 1 μM rapacuronium. Estimated [^35^S]GTPγS equilibrium binding (B_eq_) was the same for all 3 treatments (10,000 ± 300, 10,100 ± 100, and 9,980 ± 120 cpm per well for basal, ACh and ACh with rapacuronium, respectively; mean ± SE, n = 3). Rapacuronium alone slightly decreased the rate of [^35^S]GTPγS binding (Figure 7, open circles).

**Figure 8:**
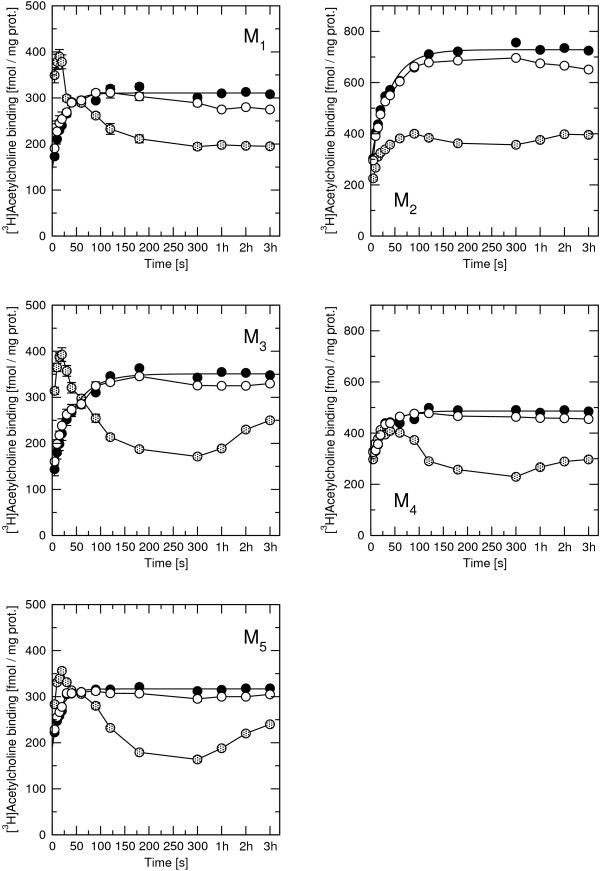
**Effects of rapacuronium on [^3^H]ACh association**. Membranes from CHO cells expressing individual subtypes of muscarinic receptors were preincubated 60 min with buffer (closed circles) or 1 μM (open circles) or 10 μM (hatched circles) rapacuronium and then [^3^H]ACh was added to a final concentration of 40 nM at time 0. Incubations were terminated at the times indicated on the x-axis. Specific [^3^H]ACh binding is expressed as fmol per mg of proteins. Data are means ± SE of values from 3 independent experiments performed in quadruplicates. Binding parameters are shown in Table 6.

Effects of rapacuronium on the association rate of high-affinity (40 nM) [^3^H]ACh binding were measured after 60 min preincubation of membranes with rapacuronium. Association of [^3^H]ACh is complex and consists of an initial very fast step in the range of seconds followed by a slower phase (Figure 8, first time point is 5 s). Under control conditions (Figure 8, closed circles) the slower phase of [^3^H]ACh association was characterized by an observed rate (k_obs_) in the range of 1.43 (M_3_) to 3.5 (M_5_) min^-1^. While the presence of 1 μM rapacuronium had marginal effects on [^3^H]ACh association (Figure 8, open circles) the association binding curve became more complex and showed a peak in the presence of 10 μM rapacuronium (Figure 8, hatched circles). This peak occurred at 15 to 20 seconds at odd-numbered receptors and at 30 to 50 seconds at the M_4 _and around 90 seconds at the M_2 _receptor. Peak binding was higher than control binding at odd-numbered receptors, the same as control binding at M_4 _and lower than control binding at M_2 _receptor. An increase in [^3^H]ACh binding after extended incubation (hours) occurred at M_3_, M_4_, and M_5 _receptors.

**Figure 9:**
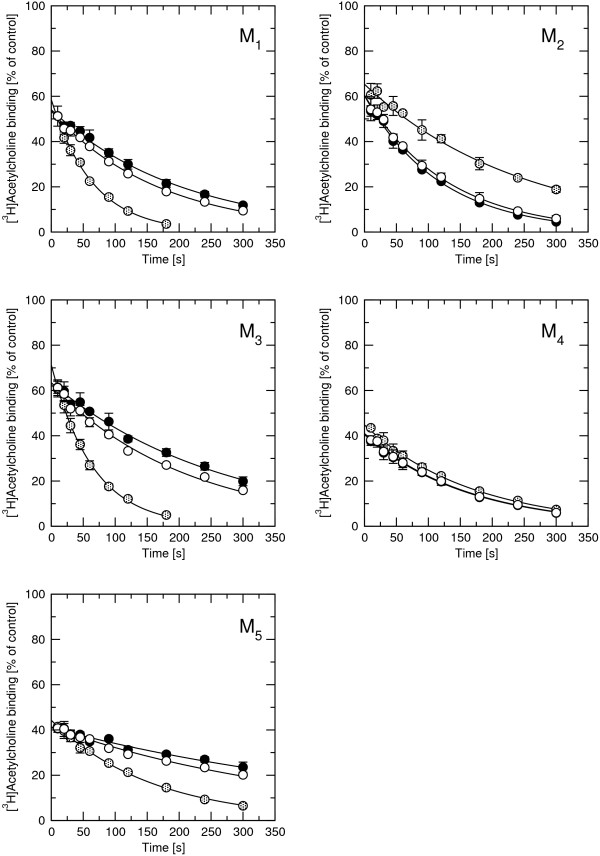
**Effect of rapacuronium on the time course of [^3^H]ACh dissociation**. Membranes from CHO cells expressing individual subtypes of muscarinic receptors were prelabeled with 40 nM [^3^H]ACh for 60 min. At time zero 40 μM unlabeled ACh was added alone (closed circles) or as a mixture with 1 μM rapacuronium (open circles) or 10 μM rapacuronium (hatched circles). Incubations were terminated at the times indicated on the x-axis. Specific [^3^H]ACh binding is expressed as percent of specific binding at time 0 on x-axis. Data are means ± SE of values from 3 independent experiments performed in quadruplicates. Binding parameters are shown in Table 6.

Effects of 10 μM rapacuronium on the dissociation rate of high-affinity [^3^H]ACh binding were measured after 60 min preincubation of membranes with 40 nM [^3^H]ACh. Dissociation was evoked by the addition of unlabeled ACh at a final concentration of 40 μM, either alone or mixed with 10 μM rapacuronium (Figure 9, Table 6). [^3^H]ACh dissociation curves consisted of a very rapid phase followed by a slow one, both in the absence (Figure 9, closed circles) and in the presence (Figure 9, open and hatched circles) of rapacuronium. The slower phase of [^3^H]ACh dissociation displayed a rate (k_off_) in the range of 0.112 (M_5_) to 0.507 (M_2_) min^-1 ^(Figure 9, closed circles). While effects of 1 μM rapacuronium on [^3^H]ACh dissociation were marginal, 10 μM rapacuronium either accelerated (odd-numbered), did not change (M_4_) or slowed (M_2_) the rate of [^3^H]ACh dissociation (Figure 9, hatched circles).

**Table 6:** Effects of rapacuronium on the rate of [^3^H]ACh association and dissociation

	40 nM [^3^H]ACh		+ 10 μM rapacuronium
	k_obs _[min^-1^]	k_off _[min^-1^]	k_off _[min^-1^]
M_1_	2.74 ± 0.25	0.294 ± 0.015	0.917 ± 0.046*
M_2_	1.58 ± 0.14	0.507 ± 0.025	0.245 ± 0.012*
M_3_	1.43 ± 0.13	0.226 ± 0.011	0.923 ± 0.046*
M_4_	2.01 ± 0.18	0.373 ± 0.019	0.355 ± 0.018
M_5_	3.50 ± 0.31	0.112 ± 0.006	0.378 ± 0.019*

Observed rates of association (k_obs_) of 40 nM [^3^H]ACh with and dissociation (k_off_) from individual subtypes of muscarinic receptors were obtained by fitting Eq. 6 to data in Figure 8 and Eq. 7c to data in Figure 9. Values are means ± SE of fits to 3 independent experiments performed in quadruplicates. *P < 0.05; significantly different from control ([^3^H]ACh alone) by t-test.

**Figure 10:**
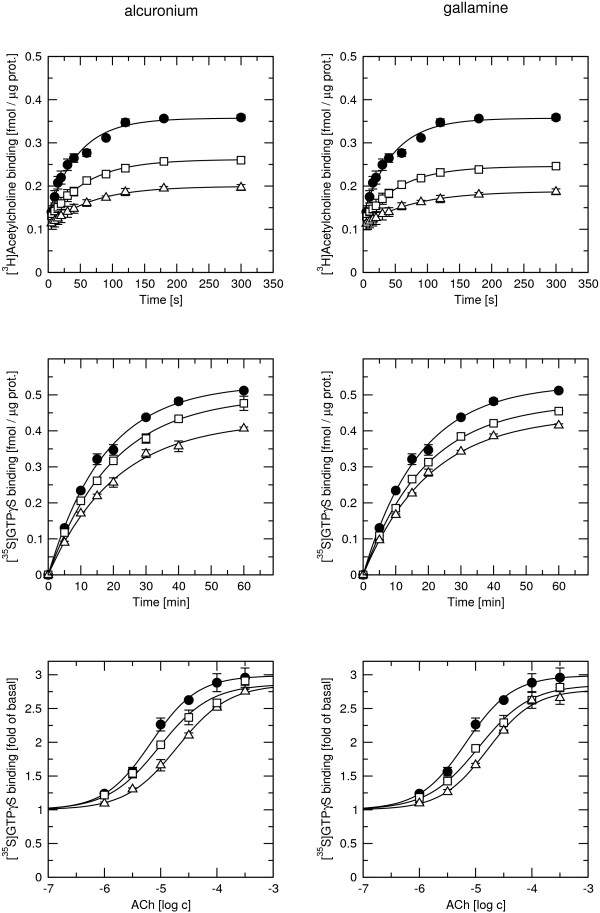
**Effects of alcuronium and gallamine on [^3^H]ACh binding and ACh-stimulated [^35^S]GTPγS binding to M_3 _membranes**. Effects of the reference allosteric modulators alcuronium (left) and gallamine (right) on kinetics of [^3^H]ACh binding (top) and ACh-stimulated [^35^S]GTPγS binding (middle) and concentration response of [^35^S]GTPγS binding to ACh stimulation (bottom) at M_3 _receptors were measured after preincubation of membranes for 60 min with buffer (closed circles) or with 1 μM (open squares) or 10 μM (open triangles) rapacuronium. Then either [^3^H]ACh (top) or [^35^S]GTPγS simultaneously with 10 μM ACh (middle and bottom) was added. Incubation was terminated at the times indicated on the x-axis (top and middle) or after 20 min (bottom). Binding is expressed as fmol per μg of protein of specific [^3^H]ACh (top) or [^35^S]GTPγS (middle) binding or as fold increase of basal [^35^S]GTPγS binding (bottom). Data are means ± SE from 3 to 6 independent experiments performed in quadruplicates. Parameters are summarized in Table 7.

The effects of rapacuronium and the two prototypic allosteric modulators alcuronium and gallamine on ACh-stimulated [^35^S]GTPγS binding and kinetics of [^3^H]ACh binding were compared at M_3 _receptors where the effects of rapacuronium were most pronounced. Measurements of ACh-stimulated [^35^S]GTPγS binding in the presence of 1 and 10 μM alcuronium or 1 and 10 μM gallamine, showed a small concentration-dependent increase in the EC_50 _of ACh without change in E_MAX _values (Figure 10, Table 7). Both alcuronium and gallamine concentration dependently slowed [^35^S]GTPγS association stimulated by 10 μM ACh. At 10 μM concentrations they also decreased [^35^S]GTPγS equilibrium binding (Figure 10, Table 7). Alcuronium and gallamine slowed down association of 40 nM [^3^H]ACh and decreased its equilibrium binding at M_3 _receptors (Figure 10, Table 7).

**Table 7:** Effects of alcuronium and gallamine on ACh-stimulated [^35^S]GTPγS binding at M_3 _receptors.

	40 nM [^3^H]ACh binding			[^35^S]GTPγS binding		
	k_obs _[min^-1^]	B_eq _[fmol/mg prot.]	k_obs _[min^-1^]	B_eq _[fmol/μg prot.]	pEC_50_	E_MAX _[fold over basal]
Control	1.28 ± 0.06	357 ± 17	0.0579 ± 0.0013	530 ± 37	5.18 ± 0.03	2.94 ± 0.12
+ 1 μM alcuronium	1.00 ± 0.02*	262 ± 10*	0. 0509 ± 0.0017*	496 ± 33	5.03 ± 0.02*	2.90 ± 0.14
+ 10 μM alcuronium	0.888 ± 0.012*	199 ± 12*	0.0481 ± 0.0021*	428 ± 39*	4.82 ± 0.02*	2.87 ± 0.12
+ 1 μM gallamine	1.06 ± 0.02*	246 ± 10*	0.0525 ± 0.0015*	479 ± 29	5.01 ± 0.04*	2.83 ± 0.12
+ 10 μM gallamine	0.783 ± 0.009*	191 ± 8*	0.0492 ± 0.0015*	442 ± 27*	4.82 ± 0.01*	2.73 ± 0.15

Values of observed rates of association (k_obs_) and equilibrium binding (B_eq_) of 40 nM [^3^H]ACh with M_3 _receptors were obtained by fitting Eq. 6 to data in Figure 10 (top). Values of k_obs _and B_eq _of 200 pM [^35^S]GTPγS with M_3 _receptors were obtained by fitting Eq. 6 to data in Figure 10 (middle). Negative logarithms of half effective concentrations (pEC_50_) and maximum stimulatory effect (E_MAX_) of acetylcholine on [^35^S]GTPγS binding in the absence or presence of the indicated concentrations of alcuronium or gallamine were obtained by fitting Eq. 5 to the data in Figure 10 (bottom). Values are means ± SE of fits to 3 to 6 independent experiments performed in quadruplicates. *P < 0.05; significantly different from control (ACh alone) by ANOVA and Tukey-Kramer post-test.

## Discussion

Our results clearly demonstrate that the neuromuscular blocker rapacuronium binds to all muscarinic receptor subtypes at physiologically relevant concentrations [18] and displays micromolar affinity and slight selectivity towards M_2 _receptor. This selectivity is smaller than that of other neuromuscular blockers such as alcuronium, gallamine and pancuronium [23, 24, Jakubík, unpublished data]. Like the majority of this class of compounds, rapacuronium acts as a negative allosteric modulator (alters dissociation kinetics and incompletely inhibits binding of orthosteric ligands) with respect to binding of both the natural agonist ACh (Figures 1, 4, 5, 8 and 9) and the classical antagonist NMS (Figures 1, 3, and 5). Rapacuronium exhibits complex effects on the kinetics of ACh binding (Figures 8 and 9) and subsequent receptor activation estimated from stimulation of [^35^S]GTPγS binding (Figures 6 and 7). Functional effects differ from those of the prototypic negative allosteric modulators alcuronium and gallamine (Figure 10, Table 7).

Our observation of an allosteric mode of interaction between rapacuronium and muscarinic receptors is in agreement with reported slowing-down of NMS dissociation from M_2 _and M_3 _receptors by this drug [19]. The observed biphasic dissociation of NMS under non-equilibrium conditions in the presence of an allosteric modulator such as rapacuronium was described earlier [24].

### Inverse receptor agonism by rapacuronium

Rapacuronium alone decreases [^35^S]GTPγS binding. This effect is mediated by muscarinic receptors because it is not observed in membranes prepared from a native CHO cell line that does not express muscarinic receptors and thus cannot be explained by nonspecific effects on cell membranes. Instead, this effect can be related to an inverse agonistic effect of rapacuronium itself on constitutive receptor activity. This view is supported by previous demonstration of constitutive activity of muscarinic receptors [25,26] and by finding that the orthosteric antagonists NMS and atropine also decrease [^35^S]GTPγS binding when applied alone [22,27]. In addition, both agonistic and inverse agonistic effects of allosteric modulators have already been observed [27,28].

### Allosteric modulation of receptor activation by rapacuronium

Both [^3^H]ACh saturation binding experiments (Figure 1) and ACh vs. [^3^H]NMS competition experiments (Figure 2) show ACh high affinity binding in the nanomolar range without selectivity towards any of muscarinic receptor subtypes. The affinities of ACh at M_2 _and M_4 _receptors reported in this study are within the range of published values, being lower than those published by Lazareno et al. [11] but higher than the values reported by Haga et al. [29] or Gurwitz et al. [30]. This divergence is likely due to the dependence of the affinity of acetylcholine at its high-affinity site on many factors (e.g. receptor source, preparation, concentration of ions (mainly Mg^2+^, Na^+^), residual concentration of GDP, temperature, etc.). Similarly, we found no subtype differences in ACh low affinity binding, which is in accordance with our previous studies [21]. Despite lack of binding selectivity, the potency and efficacy of ACh in stimulating [^35^S]GTPγS binding are significantly higher at even-numbered than at odd-numbered subtypes. In other words, the M_2 _and M_4 _subtypes that preferentially couple with G_i/o _G-proteins display better coupling and larger receptor reserve than the M_1_, M_3_, and M_5 _subtypes that preferentially couple with G_q/11 _G-proteins. Despite accumulating evidence for the existence of agonist-specific conformations of muscarinic and other G-protein-coupled receptors [31-33] it is generally accepted that the change in agonist potency in receptor activation follows a change in the affinity of its binding induced by an allosteric modulator. Thus, negative cooperativity between the allosteric modulator and the binding of an orthosteric agonist would lead to lower potency of agonist (e.g. pioneering experiments with gallamine of Clark and Mitchelson [12]) and positive cooperativity would result in higher potency of agonist [11,34]. Rapacuronium behaves in accordance with this view in case of the M_2 _and M_4 _subtypes. However, at the M_1_, M_3 _and M_5 _receptor subtypes, rapacuronium up to a concentration of 10 μM either increases or does not alter ACh potency or efficacy in inducing [^35^S]GTPγS binding (Figure 6), despite clear negative cooperativity with ACh binding (Figures 4 and 5). Although this observation may appear surprising at first glance it is perfectly in agreement with the hypothesis of multiple receptor conformations induced by orthosteric and allosteric ligands, and with the existence of conformations that exhibit low affinity for agonist binding but nevertheless activate second messenger pathways [26,31,35,36].

### Kinetics of functional response

Analysis of the kinetics of [^35^S]GTPγS binding shows that the facilitatory effects of rapacuronium on ACh-induced responses are evident after brief incubations (lasting minutes, Figure 7). This suggests that the facilitating effects of rapacuronium on ACh-induced response are a consequence of altered receptor kinetics rather than a change in agonist affinity at equilibrium. Extended time of incubation during which binding of ligands equilibrates may thus obscure the initial transient potentiation. Analysis of kinetics of ACh binding (Figures 8 and 9) showed that rapacuronium affects ACh kinetics differently than those of NMS. While rapacuronium slows down NMS association and dissociation at all receptor subtypes (Figure 3) it accelerates ACh association and dissociation at odd-numbered subtypes (Figures 8 and 9). Thus, rapacuronium doubles the magnitude of ACh binding at 15 seconds at these receptors such that association after 15 seconds is twice as much in the presence of rapacuronium. This effect, however, is counterbalanced by accelerated dissociation, resulting in an overall decrease in ACh affinity (negative cooperativity). Although combination of negative binding cooperativity on the one hand and acceleration of binding on the other could in principle be interpreted within the frame of the ternary receptor model. However, data of association and dissociation of ACh in the absence of rapacuronium do not conform to a simple bi-molecular interaction. As a result, the interaction between ACh and rapacuronium at muscarinic receptors is more complex and may involve allosteric extension of the tandem two-site model [37,38]. Theoretically, this extension of the model allows for coexistence of positive cooperativity between rapacuronium and the initial step of ACh binding and overall negative binding cooperativity under equilibrium. An enigmatic feature of our data, however, is that low concentrations of rapacuronium (0.1 and 1 μM) that do not affect the the rate of binding of ACh or its affinity at equilibrium at odd numbered subtypes leads to an increase in both potency and efficacy of ACh in receptor activation. Theoreticaly, allosteric extension of tandem two-site model allows for positive cooperativity between rapacuronium and ACh initial binding step in overall negative binding cooperativity under equilibrium and transient binding of these sub-threshold concentrations. However, these concentrations of rapacuronium had no effect on ACh association in binding experiments (Figure 8, open circles). One possible explanation is that ACh bound to a peripheral site (of tandem two-site binding) is lost during filtration but is well reflected and amplified in GTPγS binding that is pseudo-irreversible. A more speculative explanation assumes that rapacuronium at sub-micromolar concentrations binds to another site on the receptor and facilitates receptor activation by ACh without significant interference with radioligand binding. This facilitatory effect is overcome at high concentrations of rapacuronium by negative cooperativity in binding of ACh induced by binding of rapacuronium to an allosteric binding site. A latent further increase in ACh binding after 5 min in the presence of 10 μM rapacuronium (Figure 8, hatched circles) suggests an even more complex mechanism of interaction of rapacuronium wih the receptor.

Modeling of such complex kinetics would require a model even more sophisticated than ternary extension of the tandem-two site model [38]. Additionally, differential effects of low concentrations of rapacuronium (1 μM and lower) on receptor binding and function would require inclusion of receptor activation (probably with several ligand-specific activation states) in the model and therefore renders modeling unachievable.

Comparison of the effects of rapacuronium with those of the prototypic allosteric modulators alcuronium and gallamine (Figures eleven and twelve) on M_3 _receptors shows that acceleration of ACh kinetics is unique to rapacuronium among negative allosteric modulators. To our knowledge this is the first report of acceleration of binding of an orthosteric ligand by a negative allosteric modulator. This highlights unpredictability of kinetics of allosteric modulation based on compounds with similar behavior observed under equilibrium.

### Physiological implications

Our observations are consistent with functional ex vivo and in vivo physiological experiments demonstrating an increase of acetylcholine-evoked muscle contraction of guinea pig trachea rings by rapacuronium [18-20]. Although they confirm proposed allostetic interaction between rapacuronium and ACh [19] they do not conform to the proposed positive binding cooperativity at the M_3 _receptor subtype. Although rapacuronium at concentrations below 10 μM binds to and decreases the affinity of acetylcholine at equilibrium at all subtypes of muscarinic receptors, it accelerates association of ACh and enhances its potency and efficacy in functional responses at the M_3 _receptor as evident from [^35^S]GTPγS binding. The initial acceleration of the rate of association of ACh would potentiate fast responses such as bronchial smooth muscle contractions mediated by transient actions of acetylcholine at M_3 _receptors [16]. In contrast, rapacuronium at clinically relevant concentrations strongly reduces the affinity of binding of ACh and also its potency and efficacy in activating M_2 _receptors. This pattern of effects should lead to an increase in ACh release by interrupting its M_2 _receptor-mediated presynaptic autoinhibition [17] and to the inhibition of postsynaptic M_2 _receptor-mediated muscle relaxation. In contrast, the decrease of ACh affinity at the M_2 _and M_4 _subtypes is accompanied by a decrease in both potency and efficacy of stimulating [^35^S]GTPγS binding. The synergistic effects of negative functional modulation of pre- and postsynaptic M_2 _receptors and positive functional modulation of postsynaptic M_3 _receptors can explain the fatal bronchospasm caused by rapacuronium in human.

## Conclusions

Although rapacuronium exerts negative cooperativity with binding of ACh to all muscarinic receptor subtypes at equilibrium it accelerates the rate of ACh binding at odd numbered subtypes. At concentrations below 10 μM, it increases the potency and efficacy of ACh in increasing the rate of [^35^S]GTPγS binding at odd-numbered subtypes. The time between acetylcholine release and termination of its action by acetylcholinesterase is in the range of a fraction of a second. Therefore, the effects of allosteric modulators in the early non-equilibrium stage of receptor signaling are therapeutically more important than effects on acetylcholine equilibrium binding, as the latter conditions do not occur in vivo. Our study demonstrates a case of dichotomous effects of the allosteric modulator rapacuronium on ACh equilibrium binding on the one hand and on the kinetics of ACh binding on the other. Our observations emphasize the necessity to employ fast functional assays in screening for potential allosteric modulators of neurotransmission that much better simulate physiological conditions than long-lasting equilibrium binding experiments.

## Methods

### Materials

The radioligands [^3^H]-N-methylscopolamine chloride ([^3^H]NMS) and guanosine-5'-γ[^35^S]thiotrisphosphate ([^35^S]GTPγS) were from Amersham (UK), [methyl-^3^H]acetylcholine iodide ([^3^H]ACh) was from ARC (St. Louis, MO). Carbachol, dithiotreitol, gallamine triethiodide (TLC >98%), guanosine-5'-bis-phosphate (GDP), guanosine-5'-γS-thiotrisphosphate (GTPγS), and N-methylscopolamine chloride (NMS) were from Sigma (St. Louis, MO). Rapacuronium (Organon, West Range, NJ) was kindly provided by Prof. Emala, Columbia University, New York, NY. Alcuronium was kindly provided by F. Hoffmann-la Roche Ltd., Basle, Switzerland. CHO cells stably expressing individual subtypes of muscarinic receptors were provided by Dr. T.I. Bonner (National Institutes of Health, Bethesda, MD).

### Cell culture and membrane preparation

Chinese hamster ovary cells stably transfected with the human M_1 _to M_5 _muscarinic receptor genes were grown to confluence in 75 cm^2 ^flasks in Dulbecco's modified Eagle's medium supplemented with 10% fetal bovine serum and 2 million cells were subcultured to 100 mm Petri dishes. Medium was supplemented with 5 mM butyrate for last 24 hours of cultivation. Cells were detached by mild trypsinization on day 5 after subculture. Detached cells were washed twice in 50 ml of phosphate-buffered saline and 3 min centrifugation at 250 × g. Washed cells were diluted in ice cold homogenization medium (100 mM NaCl, 20 mM Na-HEPES, 10 mM EDTA; pH = 7.4) and homogenized on ice by two 30 s strokes using Polytron homogenizer (Ultra-Turrax; Janke & Kunkel GmbH & Co. KG, IKA-Labortechnik, Staufen, Germany) with a 30 s pause between strokes. Cell homogenates were centrifuged for 30 min at 30,000 × g. Supernatants were discarded, pellets resuspended in fresh incubation medium (100 mM NaCl, 20 mM Na-HEPES, 10 mM MgCl_2_; pH = 7.4) and centrifuged again. Resulting membrane pellets were kept at -20°C until assayed, for 10 weeks at maximum.

### Radioligand binding

All radioligand binding experiments were carried out on membranes in 96-well plates at 30°C in the incubation medium described above supplemented with freshly prepared dithiothreitol at a final concentration of 1 mM, essentially as described by Jakubík et al. [23]. Membranes at concentrations 4, 50 and 100 μg of protein per well were used for [^35^S]GTPγS, [^3^H]NMS and [^3^H]ACh binding, respectively. Final volume was 200 μl, except for [^3^H]NMS saturation binding that was done in 0.8 ml volume. High affinity acetylcholine and NMS binding was measured directly using [^3^H]ACh and [^3^H]NMS, respectively. Low affinity acetylcholine binding to muscarinic receptors was determined by the ability of unlabeled ACh to decrease binding of 1 nM [^3^H]NMS in the presence of 10 μM GTPγS. Nonspecific binding was determined in the presence of 10 μM NMS. Incubation with [^3^H]ACh or [^3^H]NMS lasted 60 min and was terminated by fast filtration and washing with ice cold water through Whatman GF/F glass-fiber filters (Whatman) using a Tomtech Mach III cell harvester (Perkin Elmer, USA). Filtration and washing lasted 4 s for [^3^H]ACh and 9 s for [^3^H]NMS (wash-aspirate button times). Six concentrations of [^3^H]NMS were used in saturation experiments (68 pM to 2000 pM [^3^H]NMS in the absence of rapacuronium and 189 pM to 5000 pM [^3^H]NMS in its presence). Corresponding concentrations of [^3^H]ACh were 3.4 nM to 100 nM [^3^H]ACh in the absence of paracuronium and 7 nM to 200 nM [^3^H]ACh in its presence. Effects of rapacuronium on acetylcholine high affinity binding was measured as a change in [^3^H]acetylcholine binding after 60 min prelabeling with 20 nM [^3^H]ACh followed by addition of rapacuronium and incubation for additional 3 hours. Effects of rapacuronium on ACh low affinity binding was determined as a change in [^3^H]NMS binding in the presence of 10 μM GTPγS after 60 min prelabeling of membranes with 1 nM [^3^H]NMS followed by addition of ACh with or without rapacuronium and additional 3 hours of incubation. Effects of rapacuronium on [^3^H]ACh association was measured after 60 min preincubation with 10 μM rapacuronium. When kinetics of association were measured Bio-Tek μFill (Bio-Tek Instruments, Winooski, VT) was programmed for addition of hot ligand at appropriate times before filtration. Effects of rapacuronium on dissociation of [^3^H]NMS or [^3^H]ACh binding was measured by addition of 10 or 100 μM rapacuronium to a mixture with unlabeled ligand (10 μM NMS or 40 μM ACh) to initiate dissociation.

For determination of [^35^S]GTPγS binding to G-proteins in membranes a final concentration of 200 pM (M_1_, M_3 _and M_5 _receptors) or 500 pM (M_2 _and M_4 _receptors) of [^35^S]GTPγS was used. Incubation medium was supplemented with 5 μM (M_1_, M_3 _and M_5 _receptors) or 50 μM (M_2 _and M_4 _receptors) GDP. Nonspecific binding was determined in the presence of 1 μM unlabeled GTPγS. When effects of rapacuronium on ACh-stimulated [^35^S]GTPγS binding was measured rapacuronium was added to membranes 60 min prior to ACh and [^35^S]GTPγS. Incubation with [^35^S]GTPγS was carried out for 20 min and free ligand was removed by filtration as described above. Filtration and washing with ice-cold water lasted for 9 s (wash-aspirate button time).

After filtration filters were dried in vacuum for 1 h while heated at 80°C and then solid scintillator Meltilex A was melted on filters (105°C, 90 s) using a hot plate. After cooling the filters were counted using a Wallac Microbeta scintillation counter.

### Data analysis

In general binding data were analyzed as described in Jakubík et al. [21]. Data were preprocessed by Open Office 3.0 http://www.openoffice.org and subsequently analyzed by Grace 5.1.18 http://plazma-gate.weizman.ac.il/ and statistics package R http://www.r-project.org on Mandriva distribution of Linux.

The following equations were fitted to data:

Saturation of radioligand binding(1)

y, binding of radioligand ([^3^H]NMS or [^3^H]acetylcholine) at free radioligand (after correction for depletion) concentration x; B_MAX_, maximum binding capacity; K_D_, equilibrium dissociation constant.

Competition with [^3^H]NMS binding(2)

y, binding of [^3^H]NMS at a concentration of displacer x normalized to binding in the absence of displacer; IC_50_, concentration causing 50% decrease in binding.

Equilibrium dissociation constant of displacer (K_i_) was calculated according Cheng and Prusoff [39].

Allosteric interaction between rapacuronium and [^3^H]NMS or [^3^H]acetylcholine high affinity binding(3)

y, binding of radioligand ([^3^H]NMS or [^3^H]acetylcholine) in the presence of rapacuronium at concentration x normalized to the absence of rapacuronium; [L] concentration of radioligand; K_D_, equilibrium dissociation constant of radioligand; K_A_; equilibrium dissociation constant of rapacuronium; α, factor of cooperativity between radioligand and rapacuronium [40]. Cooperativity factor greater than 1 denotes negative cooperativity and less than 1 positive cooperativity. Due to its log-normal error distribution factors of cooperativity are expressed as negative logarithms (pα) through the manuscript so negative values denotes negative cooperativity and positive value denotes positive cooperativity.

Allosteric interaction between rapacuronium and acetylcholine low affinity binding(4)

y, binding of [^3^H]NMS in the presence of rapacuronium at concentration x normalized to the absence of rapacuronium; [N] concentration of [^3^H]NMS; K_D_, equilibrium dissociation constant of [^3^H]NMS; [A], concentration of acetylcholine; K_I_, equilibrium dissociation constant form Eq. 2; K_A_; equilibrium dissociation constant of rapacuronium from Eq. 3; α, factor of cooperativity between [^3^H]NMS and rapacuronium from Eq. 3; β, factor of cooperativity between rapacuronium and acetylcholine [21].

Concentration-response(5)

y, radioactivity in the presence of agonist at concentration x normalized to radioactivity in the absence of agonist; E_MAX_, maximal increase by agonist; EC_50_, concentration of agonist producing 50% of maximal effect; nH, Hill coefficient.

Time course of association(6)

y, radioligand binding at time x; k_obs_, observed rate of association; equilibrium binding B_eq _= Bottom + Span.

Time course of dissociation(7a)

or(7b)

or(7c)

y, radioligand binding at time x normalized to binding at time 0; k_off1 _and k_off2_, dissociation rate constants; f_2_, fraction of binding site with dissociation rate constant k_off2_. When both Eq. 7a and 7b were fitted to data the better fit was chosen based on sum of squares F-test and runs test.

For fitting parameter estimates close to one expected were entered manually, parameters were constrained to reasonable range, the tolerance value was set to 0.01 and iteration steps to 30. Initial values of slope factors were always 1 constrained to 0.8 to 1.2 range.

## List of abbreviations

Ach: acetylcholine; ANOVA: analysis of variance; CHO: Chinese hamster ovary; GTPγS: guanosine 5'-O-(3-thio)triphosphate; NMS: N-methylscopolamine; TCM: ternary complex model.

## Authors' contributions

JJ carried out [^3^H]ACh and [^3^H]NMS binding studies and performed statistical analysis. AR carried out [^35^S]GTPγS binding studies. All authors participated in experimental design and draft manuscript. All authors read and approved final manuscript.

## Authors' information

**JJ **is scientist, **AR **PhD student and **VD **head of Department of Neurochenistry. **EEE **is Professor at the University of Minnesota Medical School. **JJ**, **VD **and **EEE **have decades-long experience in the research of muscarinic receptors and neurochemistry.
